# Pathogenesis of α-Synuclein in Parkinson’s Disease: From a Neuron-Glia Crosstalk Perspective

**DOI:** 10.3390/ijms232314753

**Published:** 2022-11-25

**Authors:** Shuanglong Yi, Linfang Wang, Honglei Wang, Margaret S. Ho, Shiping Zhang

**Affiliations:** School of Life Science and Technology, ShanghaiTech University, Shanghai 201210, China

**Keywords:** Parkinson’s disease, α-synuclein, glia, neuron-glia crosstalk, neuroinflammation

## Abstract

Parkinson’s disease (PD) is a progressive neurodegenerative disorder. The classical behavioral defects of PD patients involve motor symptoms such as bradykinesia, tremor, and rigidity, as well as non-motor symptoms such as anosmia, depression, and cognitive impairment. Pathologically, the progressive loss of dopaminergic (DA) neurons in the substantia nigra (SN) and the accumulation of α-synuclein (α-syn)-composed Lewy bodies (LBs) and Lewy neurites (LNs) are key hallmarks. Glia are more than mere bystanders that simply support neurons, they actively contribute to almost every aspect of neuronal development and function; glial dysregulation has been implicated in a series of neurodegenerative diseases including PD. Importantly, amounting evidence has added glial activation and neuroinflammation as new features of PD onset and progression. Thus, gaining a better understanding of glia, especially neuron-glia crosstalk, will not only provide insight into brain physiology events but also advance our knowledge of PD pathologies. This review addresses the current understanding of α-syn pathogenesis in PD, with a focus on neuron-glia crosstalk. Particularly, the transmission of α-syn between neurons and glia, α-syn-induced glial activation, and feedbacks of glial activation on DA neuron degeneration are thoroughly discussed. In addition, α-syn aggregation, iron deposition, and glial activation in regulating DA neuron ferroptosis in PD are covered. Lastly, we summarize the preclinical and clinical therapies, especially targeting glia, in PD treatments.

## 1. Introduction

### 1.1. The History and Pathogenesis of Parkinson’s Disease

Parkinson’s disease (PD) is named after the English science medical expert James Parkinson, who wrote down the first detailed description about PD in An Essay on the Shaking Palsy in 1817 [1]. With life expectancy increasing, the incidence of PD is considerably rising. There are about 7 million people in pain from PD in the world, of which the United States accounts for about 1 million [2]. In industrialized countries, the morbidity of this disease is about 0.3% and patients are mainly elder adults. For people older than 60, the morbidity sharply increases 1% every year, for people older than 80, the increase rate reached 4% [3].

**Figure 1:**
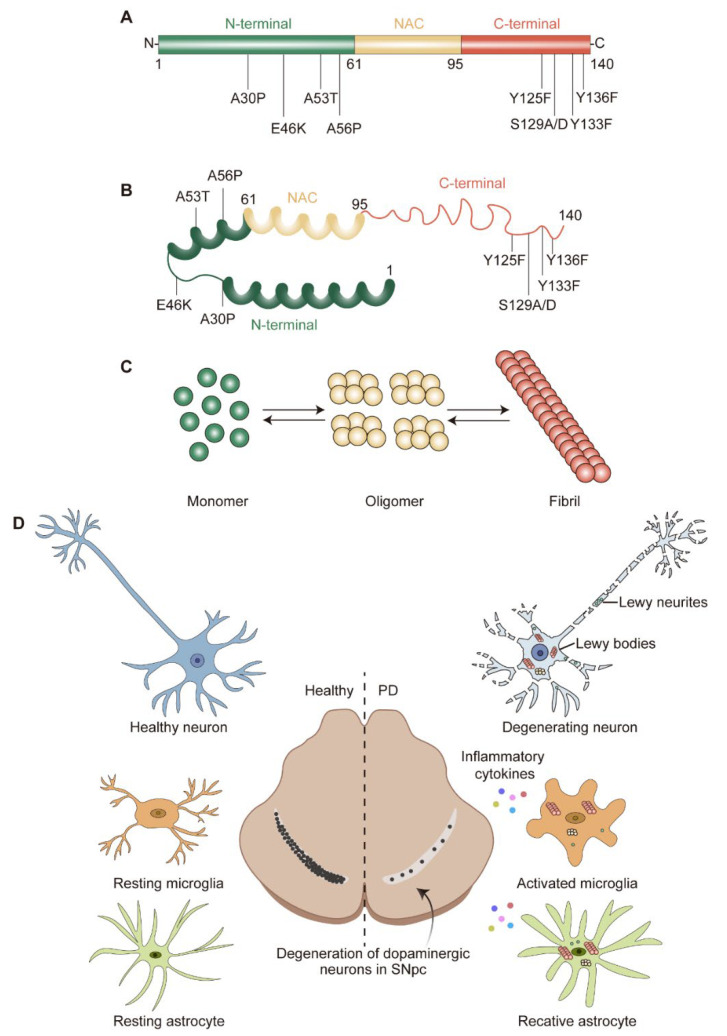
α-syn structure and pathological hallmarks of PD: (**A**) Schematic representation of α-syn. α-syn is divided into N-terminal, non-amyloid-beta component (NAC), and C-terminal; three domains highlighted in green, yellow, and red, respectively. Several familial PD-related mutations and post-translational modification sites are denoted. (**B**) Structure of α-syn monomer. (**C**) α-syn equilibrium. α-syn monomer can aggregate into oligomer or fibril. (**D**) Pathological hallmarks of PD. The pathological hallmarks of PD include progress loss of dopaminergic (DA) neurons in the substantia nigra pars compacta (SNpc), misfolded α-syn aggregates and neurites known as Lewy bodies (LBs) and Lewy neurites (LNs), and glial activation. α-syn could transfer to and activate microglia and astrocyte, which in turn release pro-/anti-inflammatory cytokines and contribute to neurodegeneration.

Upon ageing, PD patients exhibit worsening central nervous system (CNS) symptoms cultivating to defects in the motor system. The pathological hallmark of PD is the progressive loss of dopaminergic (DA) neurons in the substantia nigra pars compacta (SNpc) [4] (Figure 1D). These DA neurons are required for normal motor function, the death of which lead to bradykinesia, tremor, and rigidity [5]. Another pathological feature of PD is the formation of Lewy bodies (LBs) and Lewy neurites (LNs), which are cytoplasmic spherical protein inclusion bodies mainly composed of α-synuclein (α-syn) [6] (Figure 1D). Importantly, the spread pattern of LBs pathology correlates with the progression of PD clinical symptoms, which is also the basis of the Braak staging system [7,8]. The formation of LBs always is connected with the induction of reactive oxygen species (ROS) and the generation of superoxide radical anions, hydrogen peroxide, and hydroxyl radicals [9]. The accumulating evidence suggests that the increase in oxidative stress would exacerbate the development of PD [10].

Although the primary cause of PD cases appears to be spontaneous and widespread, most experts agree that the pathophysiology of this disease is profoundly influenced by the combination of genetic and environmental factors and that how genes and the environment interact can be very complicated. The evidence consistently suggests that a higher risk of PD is associated with a number of environmental factors, including the area of residence, occupation, exposure to metals, pesticide and herbicide exposure, and so on [11]. Accordingly, genes including *SCNA* [12], *Parkin* [13], leucine-rich repeat kinase 2 (*LRRK2*) [14], phosphatase and tensin homolog deleted on chromosome 10-induced putative kinase 1 (*PINK1*) [15], glucocerebrosidase (*GBA*) [16], vacuolar protein sorting 35 (*VPS35*) [17], and DJ-1 [18] are linked to genetic variants that directly contribute to PD. Genome-wide association studies (GWAS) also suggest that both adaptive and innate immunity may play a role in PD pathogenesis [19]. These genes help us to comprehend PD processes at cellular and molecular levels (Table 1).

### 1.2. Role of Glia in PD

Although glia outnumber neurons in the CNS, they were originally considered to be the inert “glue” (Greek “glia”) that fill in the space between neurons and play a passive supporting role due to the lack of electrical excitability [20,21,22]. Nonetheless, in the past decades, numerous studies demonstrated that glia maximize their contact with neurons and actively contribute to almost every aspect of neuronal development and function, including neurogenesis, axon guidance and ensheathment, synaptic connection and plasticity, trophic supports, elimination of dying neurons, maintaining ionic balance, and blood–brain barrier (BBB) formation [21,23,24,25,26,27,28,29,30,31]. Importantly, glial cells have been implicated in a series of neurodegenerative diseases including PD [32,33] and many PD risk genes are also expressed in glial cells [34,35,36,37], further shedding light on the importance of glia in maintaining neuronal homeostasis. Thus, gaining a better understanding of glia, especially the neuron-glia crosstalk, will not only provide insights into critical neuronal function but also advance our knowledge of neurodegenerative disease pathologies.

In the mammalian CNS, glia are classified into three different types largely based on morphological features: microglia, astrocytes, and oligodendrocytes. Microglia are the resident macrophages, serving as immune surveillants in maintaining the brain homeostasis, and playing a role in regulating the neuron circuit, synaptic pruning and plasticity, cellular debris removement, and circadian rhythms [21,38,39]. Astrocytes are large star-shaped, mostly abundant and diverse glial cells, and they regulate neuronal development, synaptic transmission, neurotransmitter recycling, trophic factors secretion, energy metabolites supplement, water and ions maintenance, and BBB formation [40,41,42]. Oligodendrocytes are the myelinating cells that generate myelin, an extended membrane that wraps tightly around axons; they are divided into two major subtypes, myelinating oligodendrocytes that can produce myelin and concentrate in white matter and nonmyelinating oligodendrocytes that concentrate in gray matter and regions with nonmyelinated axons [43].

Under physiological status, glial cells maintain the brain’s homeostasis, but under stress or pathological conditions, such as injury or increased α-syn levels, they become activated and perform detrimental or beneficial roles by triggering the secretion of inflammatory cytokines and chemokines, serving as “double-edged swords” to modulate neuropathology in PD (Figure 1D). An intracerebral injection of α-syn resulted in robust gliosis and a significant portion of α-syn inclusions were detected in astrocytes and microglia near the injection site [44]. Moreover, the widespread occurrence of α-syn inclusions in glia was observed in PD patients [45]. In human post-mortem PD brains, reactive astrocytes and microglia have been observed [46,47,48,49]. It is worthy to mention that glia function at both PD initiation when α-syn deposition occurs but no neuronal loss and PD progression when neuronal loss is evident [43].

In this manuscript, we firstly review the current understanding of α-syn structure, aggregation, and degradation in PD pathology. Then, we focus on neuron-glia crosstalk in α-syn pathogenesis, including α-syn transmission, glia activation, glial feedbacks on DA neuron degeneration, iron deposition, and DA neuronal ferroptosis. Lastly, we summarize the available PD therapies especially targeting glia.

**Table 1:** Genetic causes of PD.

Gene	Subcellular Impacts	Age of Onset	Related Mutations	Clinical Phenotypes
*BST1*	Immunity system	Late onset (>70)	rs4698412 (SNPs)	Selective vulnerability of DA neurons [50].
*HLA*	Immunity system	Late onset (>70)	HLADQA1/DQB1/DRB1 (Variant)	Increase expression of MHCII [51].
*PINK1*	Mitochondria	Young onset (20–40)	G309D, W437X, L347P, R246X	Early onset of unilateral tremor, bradykinesia, and rigidity [52].
*DJ-1*	Mitochondria	Young onset (20–40)	E64D, L166P, M26I, L10P, P158∆	Slowly progressive Parkinsonism, occasionally with behavioral or psychiatric disturbance [53].
*PARK2*	Mitochondria	~30 on average (range 16–72)	R42P, A46P, K211N, C212Y, C253Y, C289G, C441R	Parkinsonism, often presenting with dystonia, diurnal fluctuations, and sleep benefit; typically responsive to very low doses of L-DOPA [54].
*SCNA*	Endocytosis/ Autophagy	38–65 (duplications); 24–48 (triplications)	A30P, E46K, A53T, A53V	Progressive L-DOPA responsive Parkinsonism, associated with cognitive decline, autonomic dysfunction, and dementia; progression is more rapid in *SNCA* triplication cases [55].
*LRRK2*	Endocytosis	50–70 (range 32–79)	G2019S, R1441C/G/H, Y1699C, I2020T, N1437H	Parkinsonism consistent with sporadic PD; dystonia, gaze palsy, and dementia occasionally develop [56].
*VPS35*	Endocytosis	50–70 (range 34–68)	D620N	DA neuronal loss in SNpc and increase in α-syn levels [17].
*GBA*	Lysosome	45–65	N370S, L444P, K198T, R329C	Cognitive impairment, disease severity, and motor phenotype [57].
*ATP13A2*	Lysosome	Young onset (range 20–40)	T517I, A746T, S282C, R980H	Spasticity, dementia, and supranuclear gaze palsy [58].
*TMEM175*	Lysosome	Young onset (range 20–40)	M393T, K176E, G311S	Decreased glucocerebrosidase activity, facilitated α-synaggregation [59].

## 2. α-Syn Structure, Aggregation, and Degradation

### 2.1. α-Syn Structure and Physiological Function

The genetic era in PD research began in 1997, when α-syn was recognized as a key factor in this complicated neurological disease [12]. The protein α-syn is encoded by *SNCA* and consists of 140 amino acids (a.a.) with a molecular weight of approximately 15 kDa [60]. According to the physiochemical property, α-syn could be divided into three domains: a positively charged N-terminal region (1–60 a.a.) containing four regions of 11 imperfect repeats with the KTKGEV consensus sequence [61]; a central hydrophobic region (61–95 a.a.) with the non-amyloid-beta component (NAC) [62]; and a C-terminal region (96–140 a.a.) enriched with acid residues [63] (Figure 1A). α-syn is widely expressed at the presynaptic terminals of the brain [64] and regulates the vesicular transport of neurotransmitters. Under physiological conditions, α-syn is mostly found in the substantia nigra (SN), cortex, and hippocampus; it is crucial for regulating the function and plasticity of synapses [65]. Despite being highly enriched in the nervous system [64], α-syn is also expressed in a variety of other tissues, including red blood cells, heart, muscle, and other tissues with low expression levels [66], suggesting that α-syn has cellular functions beyond those specific to the nervous system.

At the cellular level, α-syn is localized to the synaptic terminal [64], mitochondria [67], endoplasmic reticulum (ER) [68], Golgi apparatus (GA) [69], endo-lysosome system [70], and also the neuronal nuclei [71]. The name synuclein combines its location in synaptic vesicles (“syn”) and nuclear envelope (“nuclein”). Up to now, there is a limited understanding about the function and physiological role of α-syn in each subcellular compartment. α-syn is linked to presynaptic terminals, sustains normal SNARE-complex assembly, controls the dopamine release [72,73], and promotes membrane curvature during synaptic vesicle budding and trafficking [64]. Increased levels of toxic α-syn cause increased mitochondrial fragmentation and decreased protein import [74,75]. When overexpressing wild-type or mutant α-syn, ER stress increases and calcium homeostasis are impaired [76]. α-syn reduces ionic transport and decreases the membrane traffic of GA [77] and deficiencies in axonal transport were linked to GA fragmentation [78]. Although the synaptic function of α-syn is well recognized [71], its role within the nucleus is less understood.

### 2.2. α-Syn Misfolding and Aggregation

Previously, PD was considered as an aging disease with an unknown specific cause or hereditary component. However, this idea was disproved in the late 1990s when *SNCA* gene variants were linked to familial, early onset forms of PD [12]. Further study provided that early onset PD are caused by duplication, triplication, and autosomal dominant missense mutations in the *SNCA* gene [79,80]. Now, it has been agreed that α-syn misfolding and subsequent aggregation contribute significantly to DA neuron degeneration in PD. This is complicated by a fast-aging global population, which coincides with an increase in the number of sporadic occurrences of PD [81,82].

Although it has long been assumed that α-syn has a natively unfolded tertiary structure [83], monomer α-syn appears to be the predominant species in the brain [84]. Alternatively, α-syn may exist as an α-helically folded tetramer [85]. Both the monomer and tetramer species are resistant to fibrillization and are present in equilibrium within healthy neurons [83,85]. Misfolded α-syn monomers could form oligomers and fibrils, which then aggregate as LBs [86] (Figure 1B,C). Particularly, the misfolding and aggregation of α-syn into fibrils depend on 71–82 a.a. in the central hydrophobic region [87]. This region can aggregate on its own and deletion of 71–82 a.a. or 66–74 a.a. prevents protein aggregation [87,88], indicating that these residues are crucial for protein misfolding and may even be the cause of amyloidosis. This feature was only observed in α-syn and as the concentration of α-syn increases, the propensity to aggregate increases.

Gene mutations in *SNCA* are also thought to promote α-syn aggregation [89]. The first specific mutation is the A53T substitution, which was an autosomal-dominant single base pair change [12]. Since then, more and more familial PD-causing autosomal dominant *SNCA* gene mutations have been identified, including A30P [90], E46K [91], H50Q [92], G51D [93], A53E [94], A53V [95], A56P [96], Y133F [97], and Y136F [97] (Figure 1A,B). While the A30P, G51D, and A53E mutations appear to slow down the rate of fibril formation, the E46K, H50Q, and A53T mutations lead to an increased rate of fibril formation. Importantly, studies on these mutants provide compelling evidence that α-syn oligomers and pre-fibrils are more toxic than mature aggregated fibrils and the aggregation of α-syn occurs in early onset PD.

The solubility and aggregation property of α-syn was also affected by post-translational modifications (PTMs), such as phosphorylation, ubiquitination, nitration, truncation, and O-GlcNAcylation [98,99]. Among these PTMs, phosphorylation at residue S129 (α-syn^pS129^) and its potential connection to α-syn-induced neurodegeneration have drawn intensive attention [63,100]. LBs in PD patient brains exists in dramatically higher amounts of α-syn^pS129^ than for normal conditions [101]. In a *Drosophila* PD model, α-syn^pS129^ was observed when expressing wild-type or mutant α-syn and the phosphorylation preferences are A53T > A30P > wild-type α-syn [102]. Further studies showed that the phosphorylation-resistant S129A mutant reduces the toxicity caused by α-syn, while the S129D mutant increases it [103]. Interestingly, other researchers also found that α-syn^pS129^ showed a reduced aggregation propensity and cytotoxicity in yeast and in vitro [104,105]. This may be explained by findings that long-range interactions could stabilize the conformation of monomeric α-syn and act as an inhibitor of oligomerization and aggregation [106], while α-syn^pS129^ could disrupt this interaction [107]. According to a recent report, α-syn at Tyr125 also can be phosphorylated (α-syn^pTyr125^) and this phosphorylation occurs at a young age but declines during the aging process. Preventing Tyr125 phosphorylation might cause α-syn to be more toxic [108]. These authors demonstrate that phosphorylation at Ser129 increases, while phosphorylation at Tyr125 decreases, the soluble oligomer of α-syn [108]. Thus, in most cases, Ser129 phosphorylation positively regulates α-syn toxicity by accelerating oligomer formation; conversely, Tyr125 phosphorylation negatively regulates α-syn toxicity by inhibiting oligomer formation.

### 2.3. α-Syn Degradation

The pathogenesis of PD and associated synucleinopathies depend heavily on the levels and conformation of α-syn. α-syn has been found to be regulated by homeostatic mechanisms via protein secretion and degradation at various points in both intracellular and transcellular ways [109]. Therefore, understanding the removal of various forms of α-syn is essential for PD pathogenesis and potential treatments.

**Figure 2:**
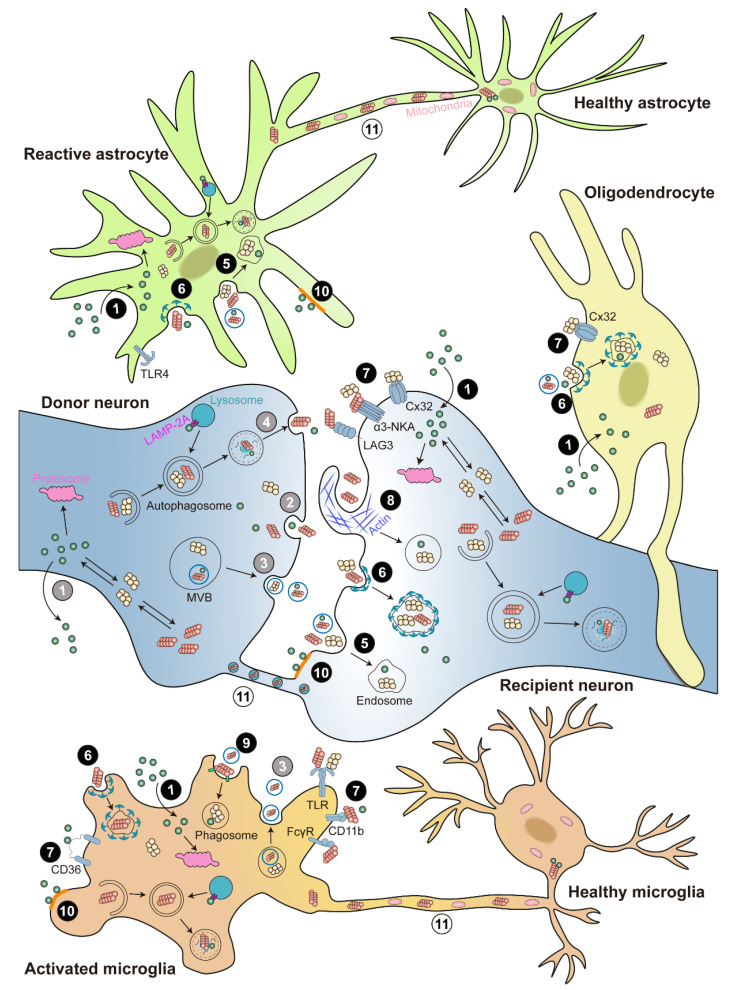
Cell-to-cell transmission of α-syn. Illustration of α-syn cell-to-cell transmission. α-syn could transfer between neurons and glia. α-syn is released via ① passive diffusion (only monomer), ② exocytosis, ③ exosomes, or ④ exophagy (grey color numbers). α-syn is taken up via ① passive diffusion (only monomer), ⑤ endocytosis, ⑥ clathrin-mediated endocytosis (CME), ⑦ receptor-mediated internalization, ⑧ micropinocytosis, ⑨ phagocytosis, or ⑩ lipid raft (black color numbers). The receptors involved in α-syn internalization include lymphocyte-activation gene 3 (LAG3), α3-subunit of Na+/K+-ATPase (α3-NKA), and the gap junction protein connexin-32 (Cx32) in neuron; Toll-like receptors 2 and 4 (TLR2 and TLR4), the scavenger receptor CD36, integrin CD11b, and the Fcγ receptors (FcγR) in microglia; and Cx32 in oligodendrocyte. In addition, α-syn can directly cell-to-cell transfer by ⑪ tunneling nanotubes (TNTs) (white color numbers). Internalized α-syn are degraded via the ubiquitin-proteasome system (UPS), autophagy, and chaperone-mediated autophagy (CMA) pathways.

Intracellular proteins are primarily degraded by proteasomal and lysosomal pathways. Proteasome degrades intracellular proteins through the ubiquitin-proteasome system (UPS), which involves the chain-like conjugation of at least four ubiquitin molecules on lysine residues of substrate proteins [110]. Lysosome degrades intracellular proteins via autophagy-lysosome pathways (ALP), including macro-autophagy (also known as autophagy), chaperone-mediated autophagy (CMA), and micro-autophagy [111]. Despite intensive studies, the exact mechanism for α-syn degradation remains controversial and varies depending on the system used. Both proteasome and lysosome were shown to be able to degrade recombinant α-syn in in vitro purified systems [112]. Accordingly, the monomeric, dimeric, pre-fibrillar, and mutant α-syn are preferentially degraded through UPS; whereas CMA degrades monomeric or dimeric α-syn, and the only way to degrade oligomeric and aggregated α-syn is autophagy. Under normal conditions, UPS is the main degradation pathway, but under stress or pathological conditions, autophagy and CMA pathways are recruited to clear the toxic α-syn burden (Figure 2).

#### 2.3.1. α-Syn Degradation through UPS

The increased accumulation of non-ubiquitinated and ubiquitinated proteins in the LBs of PD patients, as well as the reduced expression of 20S and 26S UPS subunits, suggested that proteasome was involved in the toxic manifestations of PD [110,113]. Both in vitro and in vivo studies show that α-syn is degraded by proteasomes, with not only monomeric, but also possibly pre-fibrillar α-syn species [114,115]. Due to differential effects on cellular α-syn half-life, a number of Ubi-ligases catalyze the addition of mono- or poly-Ubi chains to α-syn, with cytoprotective or toxic effects depending on the specific experimental setup [114,116,117].

The studies about the degradation of phosphorylated α-syn are contradictory. The research in cultured cells and rat primary cortical cultures revealed that the proteasome system degrades α-syn^pS129^ in a Ubi-independent pathway [118,119,120]. However, the lysosome inhibitor could lead to accumulated α-syn^pS129^ by blocking the ALP [121,122]. Using a synthesis inhibitor (cycloheximide), they found that the half-life time of α-syn^pS129^ is significantly shorter than the non-phosphorylated form, implying that the phosphorylated form is selectively targeted for degradation, and inhibition of proteasome significantly prolonged the half-life time of α-syn^pS129^ [120]. These findings suggest that monomeric α-syn^pS129^ may be degraded primarily by the proteasome and that lysosome plays a compensatory role.

Notably, multiple lines of evidence point to a possibility that elevated α-syn inhibits proteasomal activity, which could then cause an increase in α-syn levels, thus creating a pathogenic feedback loop that favors α-syn aggregation [123,124]. Understood together, only specific types of α-syn, consisting of tiny, soluble oligomers, are degraded by the UPS pathway.

#### 2.3.2. α-Syn Degradation through ALP

The role of proteasome in α-syn degradation are in debate. Investigations in numerous cellular systems found no significant accumulation of endogenous or overexpressed α-syn with proteasomal inhibition, while the inhibition or genetic depletion of proteasome in a mice model show increased and accumulated α-syn [125,126]. One possible explanation of the dispute is the assembly state and pools of α-syn exploited, since large oligomeric forms can be removed exclusively by lysosomes but not UPS [70]. According to those reports, a large fraction of α-syn were degraded via the lysosomal pathways [127,128] (Figure 2). The major evidence for autophagy in α-syn degradation comes from studies that α-syn buildup was detected after exposing cell lines to 3-methyladenine, an autophagosome formation inhibitor, when overexpressing either wild-type or mutant α-syn [112,129]. However, it is currently controversial whether aggregated, insoluble α-syn assemblies, such as those found in LBs, can be broken down by autophagy [130]. For instance, in an exogenous α-syn pre-formed fibrils (PFF) triggered endogenous an α-syn aggregation cell model, the α-syn inclusion resisted lysosomal breakdown. However, Gao and colleagues recently showed that distinct autophagy inducers resulted in the increased destruction of ingested exogenous α-syn PFF in neuronal cell lines, indicating that lysosomes may be able to remove seeded fibrillar α-syn [131]. Besides, it has been demonstrated that increasing autophagy flux upon α-syn overexpression has negative effects [129,132], including enhanced secretion of α-syn assemblies into the extracellular space, which may aid in the spreading of pathogenic α-syn, and an increase in mitochondrial degradation (mitophagy) in both cellular and animal PD models [133].

The above contradictions suggested that an alternative lysosomal mechanism might exist in α-syn degradation, as nonspecific lysosomal inhibitors had more dramatic effects than selective autophagy inhibition in some cases. This particular problem had actually been answered since the late 1980s, almost entirely through the work of Fred Dice’s lab, which confirmed the existence of CMA by a series of *C. elegans* experiments [134]. The proteins intended for CMA typically contain the targeting motif KFERQ and are selectively translocated into the autophagic pathway rather than being sequestered through bulk engulfment of cytoplasmic contents [135]. They are identified by the cytosolic chaperone heat-shock cognate 70 (Hsc70) and then transported directly into lysosomes by interaction with the lysosomes-associated membrane protein 2A (LAMP-2A) [136,137]. The involvement of CMA in α-syn degradation is supported by a number lines of evidence. The C-terminal KFERQ-like motif in α-syn could bind to LAMP-2A for CMA degradation, while for A30P and A53T mutant α-syn, although binding to LAMP-2A was not affected, they were not able to be internalized or degraded, acting as inhibitors of CMA degradation of other substrates [128,138,139]. This significant discovery has sparked curiosity in the relationship between CMA and PD pathogenesis. Lower levels of LAMP-2A were found in early-stage PD patient brains compared to healthy controls, along with an accumulation of α-syn and other CMA substrates such as myocyte-specific enhancer factor 2D (MEF2D) [128,140]. Consistently, downregulating *LAMP-2A* expression in rats results in ubiquitin-positive α-syn inclusions accumulated in SN, followed by DA neuronal death [141]. Some forms of PTMs, such as oxidation, nitration, and modification by oxidized dopamine, could hinder α-syn degradation via CMA and cause its accumulation [142]. Dopamine-modified α-syn (seen in sporadic PD patients) also obstructs the processing of other CMA substrates, very similar to A30P and A53T mutant α-syn [138,142]. Interestingly, other studies show that the complete loss of *LAMP-2A* in the murine brain did not induce significant α-syn accumulation, neither monomer nor high-molecular weight species [143]; this may result from the above-mentioned other protein degradation processes such as UPS.

## 3. α-Syn Transmission and Propagation

Although α-syn is predominantly expressed in neurons, mounting evidence proved that pathological α-syn aggregates are also found in glial cells and that the transmission of α-syn between cells could significantly accelerate neurodegeneration progression. The prevailing hypothesis for the cell-to-cell transmission of α-syn favors a “prion-like” propagation mode. Prions are self-replicating infectious protein particles that can transmit between individuals of the same, or even different, species [144]. Since experiments proving pathogenic α-syn from neurons of PD patients could transfer to healthy grafted nigral neurons [145,146], α-syn has been proposed to be a “prion-like” protein. The LBs extracts from PD patient brains are capable of inducing pathological α-syn accumulations and DA neuron degeneration in mice and macaque monkeys [147], further validating the transfer of pathological α-syn from donor to recipient neurons. Moreover, α-syn PFF “seeds” could recruit and convert endogenous soluble α-syn protein into insoluble pathological species [148,149,150]. In particular, the spreading pattern of α-syn pathology correlates to the progression of neurodegeneration and clinical symptoms [7].

As the absence of a secretory signal peptide sequence and its predominant localization in the cytosol, α-syn was initially considered to be an intracellular protein. This view was challenged by a series of experiments. The first indication that α-syn is released into the extracellular space came from the detection of this protein in the cerebrospinal fluid (CSF) and blood plasma of both PD and normal subjects [151,152]. This scenario is strengthened by observing α-syn secretion into the culture medium in various α-syn overexpression cell lines, in both monomeric and aggregated forms [153,154]. Importantly, the intravesicular α-syn is more prone to aggregate than the cytosolic protein [153]. In recent years, extracellular α-syn has gained intensive investigation due to its spreading property in disease initiation and progression, which could also be a potential therapeutic target.

How is α-syn released from the expressing neurons and taken up by the neighboring neurons or glial cells? The candidate mechanisms include but are not limited to: (1) α-syn is released via passive diffusion, exocytosis, exosomes, or exophagy; (2) α-syn is taken up via passive diffusion, endocytosis, clathrin-mediated endocytosis (CME), receptor-mediated internalization, macropinocytosis, or lipid raft; (3) Alternatively, α-syn can directly cell-to-cell transfer via tunneling nanotubes (TNTs) (Figure 2). Although multiple intercellular transmission processes have been proposed, the underlying mechanisms still need further investigation.

### 3.1. Release of α-Syn

The release of α-syn depends on its conformation, only monomeric α-syn can pass the cell membrane by passive diffusion in both directions [111]. Several active mechanisms have been proposed for the release of other α-syn conformations. It was shown that monomeric, oligomeric, and aggregated α-syn can be secreted by neuronal cells via exosomes, secreted multivesicular bodies (MVB) intraluminar vesicles (40–100 nm), in a calcium-dependent manner [115]. Compared to free α-syn oligomers, exosome-associated α-syn oligomers are more likely to be taken up by recipient cells and induce more toxicity [155]. Upon lysosomal dysfunction, which was reported in PD, exosome-mediated α-syn release and transmission were increased [156]. Importantly, the exosomal α-syn levels from PD patient plasma were higher than control groups and significantly correlate with disease severity [157].

In addition to exosomes, several unconventional ways have been identified for neurons to release α-syn. The monomeric and aggregated α-syn could be secreted via ER/Golgi-independent exocytosis in both normal and stress-induced conditions [153,158]. By impairing the autophagosome-lysosome fusion, the tubulin polymerization-promoting protein (TPPP/p25α) was shown to promote monomeric and aggregate α-syn secretion through exophagy, an exocytosis process of the autophagic α-syn intermediates. Lysosomal dysfunction and/or altered trafficking of autophagosomes results increased α-syn exophagy [159]. Rab11a, a recycling endosome regulator, was demonstrated to modulate the re-secretion of α-syn from neurons in addition to being degraded by an endosome-lysosome system [160]. It is noteworthy to mention that the above α-syn secretion routes are more extensively studied in neurons than glia (Figure 2).

### 3.2. Uptake of α-Syn

Upon α-syn release, the surrounding neurons or glial cells can take up the extracellular α-syn, a critical step for the cell-to-cell transmission of α-syn pathogenesis. The uptake mechanisms depend on not only the conformation of α-syn, but also the types of recipient cells. As discussed in the previous section, monomeric α-syn can be taken up through passive diffusion. Otherwise, the α-syn is taken up actively by endocytosis, CME, receptor-mediated internalization, macropinocytosis, or lipid raft. Once taken up by the recipient cells, pathological oligomer or fibrils α-syn would recruit the endogenous soluble α-syn and “seed” the insoluble aggregates formation [148,161,162]. Compared to neurons and astrocytes, microglia are more efficient in internalizing and degrading extracellular α-syn aggregates [163], suggesting that the uptake mechanisms are differentially regulated among cell types or that unique receptors are utilized in different cell types (Figure 2).

### 3.3. Neuron-to-Neuron Transmission of α-Syn

Both in vitro and in vivo experiments proved that neurons can take up α-syn oligomers and fibrils via CME, which was attenuated by dynamin inhibition [111,163,164,165,166]. Recently, a clathrin uncoating factor Auxilin, a homolog of the PD risk factor Cyclin-G-associated Kinase (GAK), was identified to regulate a broad spectrum of Parkinsonian-like symptoms in *Drosophila*. Reduced *auxilin* expression accelerates A30P mutant α-syn-mediated DA neuron loss, suggesting Auxilin might regulate PD progression through modulating the CME of α-syn [167]. Receptor-mediated internalization is also an important way for α-syn uptake. In a screen of transmembrane proteins for α-syn PFF binding candidates, Mao and colleagues found lymphocyte-activation gene 3 (LAG3) has the highest affinity for α-syn PFF binding but a minimal binding affinity for the α-syn monomer. In particular, neither Tau PFF nor β-amyloid oligomer or PFF bind to LAG3, suggesting that LAG3 is a specific receptor for α-syn PFF. Additionally, LAG3 is required for α-syn PFF internalization, endosomal trafficking, neurotoxicity, DA neuron loss, as well as biochemical and behavioral deficits, causing it to be an important therapeutical target to synucleinopathies [168]. The α3-subunit of Na+/K+-ATPase (α3-NKA) was identified as a cell surface partner of α-syn aggregates and their interaction strength depended on the α-syn species, with fibrils being the strongest, oligomers weak, and monomers none. Exogenous α-syn could interact with the plasma membrane, diffuse, and form clusters, trapping α3-NKA pumps and ultimately reducing the efficiency of Na+ extrusion [169]. Neurexin (1α, 2α, 1β, 2β and 3β) was also reported to be an α-syn PFF cell surface binding partner [168,169]. Besides, the gap junction protein connexin-32 (Cx32) has been implicated in the preferential uptake of oligomeric α-syn in neurons and oligodendrocytes. In cellular and transgenic mice PD models, an upregulated Cx32 protein level was observed, which correlates with α-syn accumulation. Notably, a direct interaction between α-syn and Cx32 was demonstrated in human PD cases [170]. Similar to tau fibrils, α-syn fibrils bind heparan sulfate proteoglycans (HSPGs) on the cell surface to stimulate murine C17.2 neural precursor cell uptake via macropinocytosis and transmit pathologic α-syn [171]. Macropinocytosis is a distinct fluid-phase endocytic pathway, it is characterized by actin-driven membrane ruffling that causes a rise of large endocytic macropinosomes (0.2–5 μm) [172,173]. The interaction of α-syn with the lipid raft, a specialized plasma membrane microdomain enriched in cholesterol and sphingolipids for intracellular trafficking and signal transduction [174], is crucial for its normal synaptic localization, both A30P mutant α-syn and raft disruption redistribute α-syn away from synapses [175,176], suggesting the role of the lipid raft in regulating the neuronal propagation of α-syn and the pathogenesis of PD (Figure 2).

As discussed in the previous section, the internalized α-syn oligomers or fibrils move through the endosomal pathway and target proteasome or lysosome for degradation (Figure 2). The dysregulation of the protein quality control systems results in α-syn accumulation and aggregation formation [111,164].

### 3.4. Neuron-to-Glia Transmission of α-Syn

#### 3.4.1. Neuron-to-Microglia Transmission of α-Syn

CME is also a major route for microglia to take up α-syn [163,177]. Compared to neurons and astrocytes, microglia are more efficient in internalizing and degrading extracellular α-syn aggregates, suggesting that microglia might be the major scavenger in cleaning extracellular α-syn. Moreover, the clearance depends on the activation state of microglia, since it was inhibited upon lipopolysaccharide (LPS) activation [163]. Microglia also could take up α-syn aggregates by phagocytosis [178,179,180], a form of endocytosis that engulfs large particles and occurs in professional phagocytes such as macrophages. In addition, monomeric α-syn can be internalized into microglia via monosialoganglioside (GM1)-dependent lipid rafts, which are clathrin-, caveolae-, and dynamin-independent [181], whereas α-syn aggregates enter microglia in a clathrin- and calnexin-dependent manner [177]. The knock down of *DJ-1*, a known PD risk factor, reduces cell-surface lipid raft expression in microglia and impairs their phagocytosis of soluble α-syn [182]. A series of membrane receptors are implicated in α-syn internalization and microglia activation. Toll-like receptors 2 and 4 (TLR2 and TLR4) mediate α-syn-induced microglial phagocytic activity, as well as pro-inflammatory response and ROS production [183,184]. Other involved membrane receptors including the integrin CD11b [185,186,187], the adhesion molecule CD44 [188], the protease-activated receptor 1 (PAR-1) [189], the Fcγ receptors (FcγR) [190], the macrophage antigen 1-receptor (Mac-1) [191], the microglial purinergic receptor P2X7 [192], the scavenger receptor CD36 [193], and plasma membrane ion channels [194] (Figure 2).

After being internalized into microglia, fibril, but not monomeric, α-syn is targeted to the autophagosome for degradation via selective autophagy (also known as synucleinphagy); the dysregulation of which results in α-syn accumulation and DA neuron degeneration [190,195] (Figure 2). TLR4-induced NF-κB signaling also upregulates the expression of p62/SQSTM1, an autophagy receptor, and promotes the formation of α-syn/ubiquitin-positive puncta for synucleinphagy [195]. Persistent α-syn fibril accumulation may lead to lysosomal damage, which induces autophagy by recruiting the selective autophagy-associated proteins TANK-binding kinase 1 (TBK1) and optineurin (OPTN) [196]. Moreover, DJ-1 is reported to regulate α-syn clearance in microglia, as *DJ-1* knock down (KD) exhibits impaired autophagy-dependent substrate degradation [182].

Notably, microglia-to-neuron transmission of α-syn was also observed. Primary microglia and microglial BV-2 cells also can secrete α-syn through exosomes and facilitate α-syn transmission, contributing to the progression of α-syn pathogenesis in PD [197,198] (Figure 2). Upon treating with human α-syn PFF, microglia could release α-syn-containing exosomes and induce protein aggregation in the recipient neurons; this can be further enhanced when combining with microglial pro-inflammatory cytokines. Importantly, the scenario was proved in mouse models and in the CSF of PD patients [197].

#### 3.4.2. Neuron-to-Astrocyte Transmission of α-Syn

Astrocyte is the most abundant cell type in the brain, thus the spreading of α-syn between neurons and astrocytes have been widely studied [199,200,201,202,203,204,205]. In co-culture experiments of induced pluripotent stem cell (iPSC)-derived astrocytes and ventral midbrain DA neurons (vmDAns) from familial mutant LRRK2 G2019S PD patients and healthy individuals, accumulated α-syn was observed in control astrocytes when co-cultured with PD vmDAns, demonstrating the direct neuron-to-astrocyte transfer of α-syn [202]. Conversely, accumulated α-syn was observed in control vmDAns when co-cultured with PD astrocytes, indicating the astrocyte-to-neuron transfer of α-syn [202]. Thus, there exists a bidirectional transfer of pathological α-syn between vmDAns and astrocytes. Other evidence also indicates that α-syn aggregates can transfer between neurons and/or astrocytes, but with different efficiencies, and α-syn is efficiently transferred via neuron-to-astrocyte and astrocyte-to-astrocyte, but less efficiently via astrocyte-to-neuron [200,201]. Nevertheless, astrocyte-to-neuron transfer of α-syn lead to neuronal death and morphological signs of neurodegeneration [200,202]. These findings unravel a crucial role of neuron-astrocytes crosstalk during PD pathogenesis and provide a novel path exploring therapeutic strategies.

The primary rat astrocytes, either cultured in medium containing secreted α-syn from differentiated SH-SY5Y cells or co-cultured with SH-SY5Y cells expressing human α-syn, could internalize neuronal α-syn through CME, which was further supported by a transgenic mouse model expressing human α-syn [199]. In particular, the endocytosis of α-syn aggregates in astrocytes is far more efficient than neurons [200]. In astrocytes, while TLR4 mediates α-syn-induced pro-inflammatory response and ROS production, the uptake of α-syn is not dependent on TLR4 expression, a different scenario compared with microglia [184] (Figure 2).

Different from neurons, astrocytes are able to efficiently degrade fibril α-syn, indicating a neuroprotective role for astrocytes in clearing α-syn deposits [201]. The α-syn aggregates within astrocytes are generally degraded via proteasome, autophagy, and CMA pathways; prolonged exposure to pathological α-syn lead to dysfunction of these pathways and, thus, astrocytic α-syn accumulation [201,202,203] (Figure 2). It is noteworthy to mention that selectively overexpressing nuclear factor erythroid 2-related factor (Nrf2) in astrocytes (GFAP-Nrf2) protects against neuronal A53T α-syn-mediated toxicity and motor pathology by promoting A53T α-syn degradation through the CMA and autophagy in vivo [206]. On the other hand, the overexpression of α-syn in immortalized astrocyte cell lines resulted in autophagy inhibition, loss of mitochondrial membrane potential and apoptosis, with A30P and A53T mutant α-syn showing a more intense and sustained effect [207]. In toto, these evidence highlight astrocyte as an important player in α-syn degradation and PD pathology. At the same time, astrocyte-related genes or signals, for instance the Nrf2 pathway, could serve as potential targets to develop therapeutic strategies for pathologic synucleinopathies.

#### 3.4.3. Neuron-to-Oligodendrocyte Transmission of α-Syn

In PD patients, complement-activated oligodendrocytes were revealed in SN [208] and α-syn inclusions were also found in oligodendrocytes [209]. Later investigation found only nonmyelinating oligodendrocytes are affected in the late stage of the PD process, rather than the initiation stage [43]. In a PD mouse model, a significant decrease in oligodendrocytes was observed in the striatum after the administration of MPTP (1-methyl-4-phenyl-1,2,3,6-tetrahydropridine) [210]. MPTP is a neurotoxic byproduct of synthetic heroine that can be taken up by DA neurons, when transformed into its ion form 1-methyl-4-phenylpyridinium (MPP+), it could inhibit the respiratory chain machinery complex 1 in mitochondria, initiate neuronal death, and finally induce Parkinsonian syndrome in human and animal models [211,212,213]. Besides, α-syn aggregates-constituted glial cytoplasmic inclusions (GCI) within oligodendrocytes and are the histopathological hallmark of Multiple System Atrophy (MSA), a progressive adult onset neurodegenerative disorder with synucleinopathies [214].

Oligodendrocyte take up recombinant α-syn monomers, oligomers, and, to a lesser extent, fibrils via CME in both a time- and concentration-dependent manner [166,215,216,217]. As aforementioned, Cx32 also has been implicated in the uptake of oligomeric α-syn in oligodendrocytes [170] (Figure 2). Furthermore, α-syn derived from host rat brain neurons overexpressing human α-syn can transfer to grafted oligodendrocytes, supporting a neuron-to-oligodendrocyte transfer of α-syn [217].

### 3.5. An Alternative Way for α-Syn Intercellular Transmission: TNTs

TNTs are a distinct form of cell-to-cell interaction. The F-actin- and myosin Va-driven connection channel (50–200 nm in diameter) enable the intercellular exchange and transfer of organelles, surface receptors, cytoplasmic molecules, and membrane components [218]. TNTs are found in various types of cells, the connection is not only restricted to the same cell type, they are also detected between different cell types, such as neurons-astrocytes [219,220,221], thus it may serve as an alternative way for α-syn intercellular transmission.

Misfolded α-syn fibrils were efficiently transferred from donor neuron to recipient neuron via TNTs inside lysosomal vesicles; once in the recipient neuron, they could recruit soluble α-syn and induce de novo α-syn aggregate formation; as a consequence, α-syn fibrils are able to spread and propagate from stressed neurons to unstressed neurons through TNTs [222]. Similar α-syn transfer through TNTs were observed in SH-SY5Y cells, as well as in primary brain pericytes from PD patients [223]. Astrocyte and microglia were also reported to transfer α-syn inclusion via TNTs. When stressed by lysosome degradation defects, human astrocytes actively transfer α-syn aggregates to the nearby healthy astrocytes via TNTs; rather than degrade it, the healthy astrocytes in return deliver mitochondria to the stressed astrocyte, establishing a TNT-mediated rescue mechanism [224]. Recently, it is reported that microglia, when exposed to α-syn, could establish a TNT network to transfer α-syn fibrils from overloaded microglia to neighboring healthy microglia where α-syn got rapidly and effectively degraded; the healthy microglia in turn donate mitochondria to α-syn overloaded microglia, sharing the α-syn burden attenuated the inflammatory profile of microglia and improved their survival. Moreover, the transferring efficiency was significantly compromised in microglia carrying LRRK2 G2019S, further proving the vital role of TNTs in α-syn transmission and PD progression [225] (Figure 2).

## 4. α-Syn-Induced Glial Activation

### 4.1. α-Syn-Induced Microglia Activation

In the adult brain, microglia are more than cleaners and amyloid phagocytes, they govern and modulate neuronal function and homeostasis. Reactive microglia were first detected in the SN of PD patient brains in 1988, suggesting it is a sensitive index of neuropathologic activity [49]. A series of studies in the PD animal model also proved early microglia activation upon α-syn overexpression, but the response differs depending on whether neuronal cell death was induced or not, suggesting that microglia may play different roles at different stages during PD progression [226,227,228]. α-syn-induced microglial activation is peptide-dependent, as the A53T protein activated more strongly than wild-type or other mutants [229]. Microglia are also involved in the early onset of PD, as activated microglia were observed in both presymptomatic and symptomatic mouse brains and the suppression of microglial activity extended the survival of astrocyte α-syn mutant mice [230]. In a double-mutant human α-syn overexpression PD mouse model and a natural cytotoxic compound rotenone-treated PD rat model, activated microglia and increased proinflammatory molecules were observed before neuron death occurred [231,232]. Using in vivo positron emission tomography (PET) imaging, microglial activation was observed in early PD patient brains [233,234]. Besides, either absence or hyperstimulation of microglia affects α-syn cell-to-cell transfer in a transplanted mouse PD model [235]. Understood together, these findings indicate a critical role of microglia in PD disease progression.

A growing number of evidence suggests that neuroinflammation is a crucial contributor, but not just a consequence, to the pathogenesis of PD. α-syn-triggered microglial activation may establish a chronic neuroinflammatory milieu, exacerbating pathogenic processes. In the Thy1-α-syn transgenic PD mice, as well as adeno-associated virus 2 (AAV2)-mediated delivery of human wild-type or A53T mutant α-syn into the SN of mice, α-syn overexpression caused an early microglial activation and inflammatory response [228,236,237]. Reactive microglia could induce DA neuron oxidative stress, then lead to α-syn nitration and cell death in PD models both in vitro and in vivo [191,238,239]. Thus, the neuron-microglia crosstalk forms a vicious cycle in PD and the neuronal-released α-syn activates microglia and produces proinflammatory factors and ROS, which in turn lead to persistent and progressive nigral neurodegeneration [180].

Activated microglia exhibit M1 classic/neurotoxic phenotypes or M2 alternative polarization/neuroprotective phenotypes, depending on the involved cytokines or inflammatory factors. The M1 state microglia often have an amoeboid morphology and release pro-inflammatory factors such as tumor necrosis factor-α (TNF-α), interleukin-1β (IL-1β), IL-12, and inducible nitric oxide synthase (iNOS). On the contrary, M2 state microglia have thin cellular bodies and ramified processes and secrete anti-inflammatory cytokines including TGF-β, IL-10, IL-4, IL-13, and neurotrophic insulin-like growth factor 1 (IGF-1). The pro-/anti-inflammatory state of microglia cause it to be “double-edged swords” to modulate neuron homeostasis and an impaired balance between M1 and M2 states was found in neurodegenerative diseases such as PD [179,240,241,242]. In a 1-methyl-4-phenyl-1,2,3,6-tetrahydropyridine-probenecid (MPTPp)-induced chronic PD mouse model, DA neuron degeneration was correlated with a gradual switch from the M2 to M1 phenotype and this can be converted by the treatment of rosiglitazone, a neuroprotective peroxisome-proliferator-activated receptor (PPAR)-γ agonist [243]. A similar transit from the M2 to M1 phenotype was observed with age [244]. Therefore, novel drugs or approaches that enhancing the neuroprotective M2 phenotype or promoting the M1 to M2 switch may have therapeutic feasibility for PD [241].

**Figure 3:**
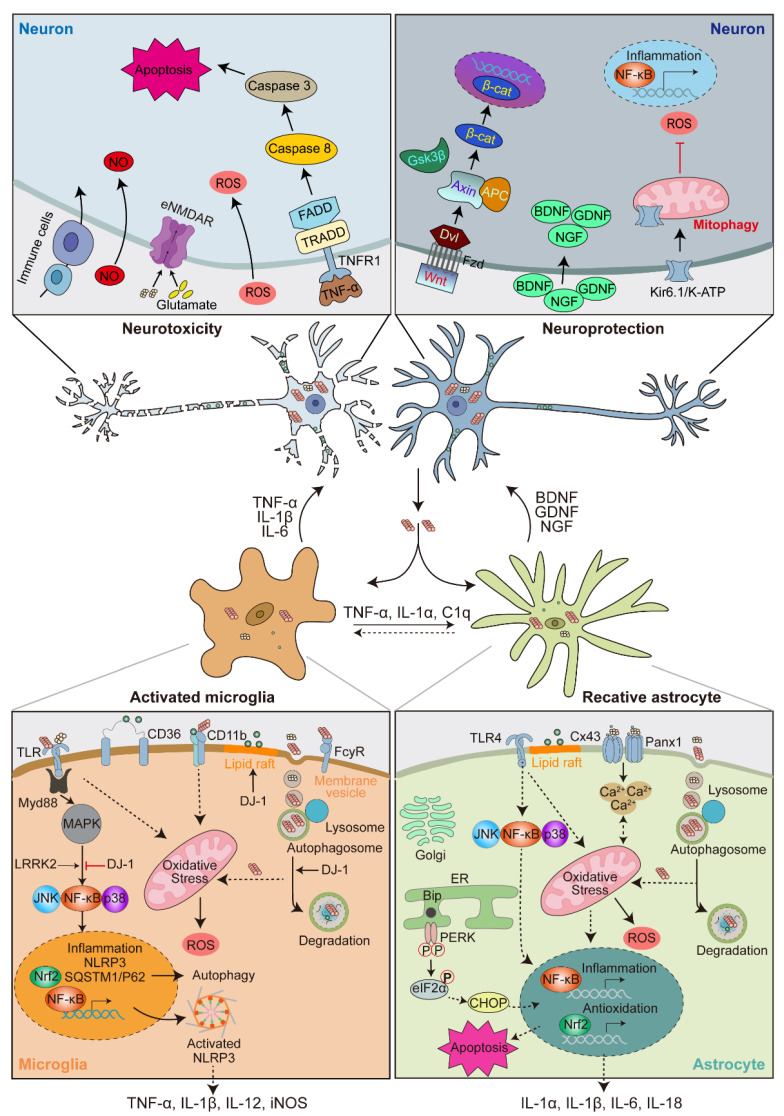
Neuron-glia crosstalk in regulating the pathogenesis of α-syn in PD. Neuronal released α-syn could transfer to and activate microglia and astrocyte, activated glia in turn perform neuroprotective or neurotoxic feedback to DA neuron. Bottom left: In microglia, uptake of α-syn triggers various signaling cascades. Upon α-syn binding to heterodimer TLR1/2 receptor, it interacts with adaptor molecule MyD88 and initiates sequential IRAK/TRAF6/TAK1 activation events, then leads to MAPK activation and nuclear translocation of NF-κB, JNK, and p38, which promote the transcription of pro-inflammatory cytokines including TNF-α, IL-1β, IL-12, and inducible nitric oxide synthase (iNOS). TLR4 mediates α-syn-induced phagocytic activity, pro-inflammatory cytokine release, and ROS production. TLR4-induced NF-κB signaling upregulates the expression of p62/SQSTM1 and promote autophagy. Nod-like receptor protein 3 (NLRP3) inflammasome is involved in the inflammatory response. DJ-1 regulates α-syn phagocytosis, inflammatory responses, and degradation in microglia. LRRK2 positively regulates NF-κB inflammation pathway. Nrf2 was also reported to be involved in α-syn-induced microglia activation. Bottom right: In astrocyte, α-syn-induced pro-inflammatory cytokines, ROS production, JNK and p38 activation, and NF-κB nuclear translocation all depend on TLR4. Monomeric and oligomeric α-syn induce a Ca^2+^ flux via opening of connexin 43 (Cx43) hemichannels and pannexin-1 (Panx1) channels. α-syn also could triggered ER stress through PERK/eIF2α signaling pathway and CHOP-mediated apoptosis pathway. Golgi fragmentation was also observed. Nrf2 plays a neuroprotective role via reducing oxidative stress. Top right: Activated glia may perform neuroprotective roles via providing trophic factors including brain-derived neurotrophic factor (BDNF), glial-derived neurotrophic factor (GDNF), and nerve growth factor (NGF). When glia are activated, the concentration of these trophic factors decreased. Astrocyte-derived factors Wnt1 could maintain DA neuron integrity via activating Frizzled (Fzd) receptor and β-catenin signaling in DA neuron, functioning as a neuroprotective pathway. The Kir6.1/K-ATP channel on astrocytes could protect against DA neuron degeneration via promoting mitophagy, which resulted in a decrease in mitochondria accumulation, ROS production, and neuroinflammation. Top left: Activated glia may perform neurotoxic roles via production of pro-inflammatory cytokines or ROS. The pro-inflammatory cytokines TNF-α may exert a more direct effect through binding to the tumor necrosis factor receptor (TNFR) expressed by DA neurons, the intracellular death domain of TNFR1 binds to the adaptor molecules TNFR1-associated protein with a death domain (TRADD) and FAS-associated protein with a death domain (FADD), then activate the apoptosis effector caspases. Pro-inflammatory cytokines can also induce glial NO and ROS production, both of which are deleterious to DA neurons. Activated microglia also lead to severe DA neuronal degeneration by promoting phagocytic exhaustion and upregulating chemotactic molecules to selectively recruit peripheral immune cells. Oligomeric α-syn induces excessive astrocytic glutamate release, which leads to further the activation of neuronal extrasynaptic NMDA receptors (eNMDARs). Meanwhile, oligomeric α-syn also directly activates eNMDAR. The aberrant eNMDAR activity finally contributes to synaptic damage and loss.

α-syn-activated microglia trigger various immune responses and signaling cascades. The injection of α-syn PFF, but not the monomer, in the SN led to microglial activation and increased major-histocompatibility complex II (MHCII) molecules both in microglial cells and in newly recruited monocytes, macrophages, and lymphocytes [245]. Upon α-syn binding to heterodimer TLR1/2 receptors, the C-terminal intracellular Toll-interleukin 1 receptor (TIR) domain interact with adaptor molecule MyD88 and initiate sequential IRAK/TRAF6/TAK1 activation events, then lead to MAPK activation and nuclear translocation of NF-κB, JNK and p38, ultimately resulting in the production of TNF-α and IL-1β pro-inflammatory cytokines [229,246,247,248]. TLR4 also mediates α-syn-induced microglial phagocytic activity, pro-inflammatory cytokine release, and ROS production [184]. Prostaglandin E2 receptor subtype 2 (EP2) was reported to play a critical role in microglial activation and associated neurotoxicity [249]. Accumulating studies proved that the nod-like receptor protein 3 (NLRP3) inflammasome is linked to activated microglia-induced inflammatory response in PD, upon α-syn stimulation, NLRP3 complex trigger caspase-1 activation and caspase-1-dependent release of IL-1β and IL-18 [250,251,252]. Some key PD risk factors are also involved in regulating the inflammatory responses of microglia. LRRK2, which is highly expressed in both neurons and microglia, is activated in response to α-syn overexpression, LPS exposures, or TLR4 stimulation, and serves as a positive regulator of the NF-κB inflammation pathway. The inhibition of LRRK2 attenuates microglial inflammatory responses and leads to DA neuron degeneration resistance [253,254,255]. In *DJ-1* KD microglia, the α-syn-induced secretion of the pro-inflammatory cytokines IL-6 and IL-1β, as well as the production of nitric oxide (NO), were elevated [182]. Another key aspect of α-syn-induced microglia activation is ROS production. In microglial BV2 cells and primary microglia, extracellular α-syn stimulates the purinergic P2X7 receptor and macrophage antigen-1 (Mac-1) receptor, leading to increased oxidative stress and cellular injuries by NADPH-oxidase, importantly, A30P or A53T mutant α-syn producing more rapid and sustained effects than wild-type α-syn [180,191,192]. In addition, Nrf2 was involved in α-syn-induced microglia activation [229,256], further investigation proved *Nrf2* deficiency cooperates with α-syn to promote protein aggregation, neuroinflammation and neuronal death, by shifting microglia from anti-inflammatory to pro-inflammatory states [257] (Figure 3).

### 4.2. α-Syn-Induced Astrocyte Activation

Since astrocytes are the most numerous glial cells, accounting for at least one third of the brain mass, and the permeability of BBB is disrupted in PD patients [258,259], it is not surprising that astrocytes α-syn inclusions are detected in different regions of PD patients and that the number of these inclusions correlates with the severity of nigral neuronal loss [46,209,260]. In Braak stages 4–6 of sporadic PD, large numbers of α-syn immunoreactive astrocytes were observed [46]. In the brain of M83 A53T α-syn Tg mice, the hippocampal delivery of α-syn fibrils resulted in α-syn^pSer129^-positive inclusions within astrocytes [44]. In PD patients and MPTP-treated monkeys, the presence of ICAM-1-positive reactive astrocytes in brain regions with heavy neuronal loss indicates a sustained astrocyte inflammatory process [261]. In rat PD models induced by the injection of 6-hydroxydopamine (6-OHDA), a catecholaminergic neurotoxin that can be taken up by DA neurons and cause nigrostriatal degeneration [213], astrocytic activation at the lesion site was also observed [262]. Notably, the astrocytes could function as the initial trigger of PD onset. When A53T mutant α-syn was selectively expressed in astrocytes, increasing α-syn aggregates were observed in both presymptomatic and symptomatic mouse brains, and these mice developed an early onset and rapidly progressed movement disability [230]. Importantly, the astrocyte α-syn was able to initiate the non-cell autonomous killing of DA and motor neurons via microglia-mediated inflammatory responses and the suppression of microglial activity extended the survival of mutant mice. These results suggested a critical role of astrocyte α-syn cytotoxicity for initiating neurodegeneration [230].

The reactive astrocytes have characteristics of hypertrophy, proliferation, and upregulated expression of glial fibrillary acidic protein (GFAP) [263]. Similar to microglia, reactive astrocytes are classified into harmful A1 astrocytes and protective A2 astrocytes, depending on their neuroprotective or neurotoxic effects. A1 astrocytes, induced by activated neuroinflammatory microglia via secreting TNF-α, IL-1α, and C1q, lost their neuroprotective ability and lead to neuronal and oligodendrocyte cell death [264]. When exposed to neuronal α-syn, astrocytes produce many pro-inflammatory cytokines (IL-1α, IL-1β, IL-6), intercellular adhesion molecule-1 (ICAM-1), and CC, CXC, and CX3C-type chemokines, with mitochondrial dysfunction and extracellular hydrogen peroxide production. It is noteworthy to mention that the inflammatory responses correlated with the extent of glial α-syn accumulation and the conformation of different α-syn species [199,265,266].

The exogenous α-syn-induced inflammatory responses depend on TLR4, since the induction of pro-inflammatory cytokines, ROS production, activation of JNK and p38, and nuclear translocation of NF-κB were all abolished in *TLR4* knockout astrocytes, however, the uptake of exogenous α-syn was unaffected [184,267]. Besides, monomeric and oligomeric α-syn induce a Ca^2+^ flux in astrocytes, as well as gliotransmitter release, NO production, and mitochondrial morphology changes, presumably leading to neurotoxicity. Further investigation in mouse cortical astrocytes indicates the altered Ca^2+^ dynamics may result from α-syn-induced opening of connexin 43 (Cx43) hemichannels and pannexin-1 (Panx1) channels [268]. In human astrocytes, α-syn treatment results in GFAP reactivity, apolipoprotein E redistribution and cholesterol reduction [269]. The overexpression of A53T and A30P mutant α-syn in astrocytes triggered ER stress through the PERK/eIF2α signaling pathway and induced the CHOP-mediated apoptosis pathway. Besides, Golgi fragmentation, decreased levels of glia derived neurotrophic factor (GDNF), and inhibited neurite outgrowth were also observed [270]. Several studies also proved the neuroprotective role of astrocyte Nrf2. Nrf2 expression in astrocytes (GFAP-Nrf2) is sufficient to protect against MPTP- or human A53T mutant α-syn-induced toxicity, motor pathology, and α-syn aggregation by promoting A53T mutant α-syn degradation through the CMA and autophagy pathway in vivo [206,271]. Moreover, the activation of endogenous Nrf2 by the primary astrocytes administration of siRNA against Kelch-like ECH associating protein 1 (Keap1) protein, a negative regulator of Nrf2, is sufficient to reduce oxidative stress and protect against MPTP-induced dopaminergic terminal damage [272] (Figure 3).

### 4.3. Microglia-Astrocyte Crosstalk

Notably, there also exist multiple crosstalk between microglia and astrocyte (Figure 3). It is well-recognized that neuroinflammatory microglia could activate A1 astrocytes via secreting TNF-α, IL-1α, and C1q [264,273]. Furthermore, blocking A1 astrocyte conversion by microglia is neuroprotective, it could prolong life and reduce neuropathological and behavioral deficits in a human A53T mutant α-syn transgenic mouse model [274]. Conversely, astrocyte, in turn, can modulate microglia activity. Astrocytic Orosomucoid-2 (ORM2) was reported to exert anti-inflammatory effects by inhibiting microglial activation and neuroinflammation [275]. While another study reported an activation effect on microglia by astrocytes in trimethyltin intoxication [276]. Specifically, when A53T mutant α-syn was expressed in mice astrocytes, reactive astrocytes and increased inflammatory responses were observed in early PD onset; at the same time, microglial activation was detected mainly in brain regions with significant DA and motor neuron loss [230]. These evidence is unraveling the intimate and complex microglia-astrocyte crosstalk and further elucidating the mechanisms that will help us to understand the PD pathologies better.

## 5. Feedback of Glial Activation on DA Neuron Degeneration

When activated by α-syn, glia may play neuroprotective roles via providing trophic factors or protecting against oxidative stress. On the other hand, glial cells may perform neurotoxic roles via the production of pro-inflammatory cytokines or ROS (Figure 3). Activated glia may perform neuroprotective or neurotoxic roles depending on the stage of PD and the conditions of glial cells such as the ROS level [277,278,279,280]. Accordingly, activated microglia and astrocyte are neuroprotective in the initial stage of PD, but become neurotoxic to promote neurodegeneration in a later stage [43,279]. In an in vivo PET imaging study of PD patients, reactive astrocytes were increased in the brainstem in the early stage of PD, while decreased in the cortex and brainstem in the middle and late stages of PD [281]. It is speculated that activated microglia and astrocyte may be initially non-toxic and neuroprotective by producing trophic factors, neurotrophins, and antioxidant substances. However, in the later stage they become neurotoxic owing to a toxic change caused by other factors such as long-term α-syn stress, toxic substances, viruses, or inflammatory cells that might occur to promote PD progression [277,279,280].

### 5.1. Neuroprotective Role of Activated Glia

Glial cells could provide trophic factors essential for DA neuron survival, among these factors, the most well-known are brain-derived neurotrophic factor (BDNF), glial-derived neurotrophic factor (GDNF), and nerve growth factor (NGF) [282,283] (Figure 3). The overexpression of α-syn results in GDNF and BDNF downregulation both in vitro and in vivo [284,285,286,287], and the activation of BDNF transcription could suppress abnormal α-syn expression and ameliorate DA neuron degeneration [284]. In PD patients and PD animal models, the concentrations of BDNF, GDNF, and NGF were significantly decreased [288,289,290]. Importantly, the injection of GDNF or NGF attenuates oxidative stress and improves dopamine function in the SN region, leading to restored motor behavior in MPTP- or 6-OHDA-induced PD animal models [291,292,293,294,295,296]. In addition, astrocyte-derived factors, including Wnt1, could maintain DA neuron integrity via activating the Frizzled (Fzd) receptor and β-catenin signaling in the DA neuron and function as a neuroprotective pathway in MPTP-induced in vitro and in vivo PD models [297,298] (Figure 3). These observations suggest that glia derived factors are involved in PD pathogenesis.

Glia cells also play neurorescue roles by relieving the oxidative stress of the DA neuron. In a rotenone-induced PD model, iPSCs-derived astrocytes can release functional mitochondria into the media, these mitochondria are then internalized by the injured DA neurons via a phospho-p38-depended pathway, thus iPSCs-derived astrocytes can reverse DA neuron degeneration and attenuate the pathology by direct mitochondrial transfer [299]. The Kir6.1/K-ATP channel on astrocytes could protect a MPTP-induced PD mouse model against DA neuron degeneration via promoting mitophagy, which resulted in a decrease in mitochondria accumulation, ROS production, and neuroinflammation [300] (Figure 3). Besides, astrocytes could remove axonal debris in a medial forebrain bundle via autophagy, preventing the spread of retrograde axonal degeneration in 6-OHDA-induced rat and mice PD models [301].

### 5.2. Neurotoxic Role of Activated Glia

Activated glial cells could modulate α-syn-induced neurotoxicity through different mechanisms. Microglia-specific overexpression of α-syn leads to severe DA neuron degeneration by promoting phagocytic exhaustion and upregulating chemotactic molecules to selectively recruit peripheral immune cells [302]. Microglia activation also promotes PD patients’ plasma-derived exosomal α-syn transmission to neurons both in vitro and in vivo [303]. The inflammatory cytokine IL-1β, released by activated glia, is able to upregulate α-syn expression in a time- and concentration-dependent manner [304]. Trudler et.al. found that oligomeric α-syn could induce neuronal damage via an astrocyte-dependent or astrocyte-independent manner. Oligomeric α-syn induces excessive astrocytic glutamate release, which leads to further activation of neuronal extrasynaptic NMDA receptors (eNMDARs). Meanwhile, oligomeric α-syn also directly activates eNMDAR. The aberrant eNMDAR activity finally contributes to synaptic damage and loss [305] (Figure 3).

Glial activation-released pro-inflammatory cytokines are deleterious to DA neurons. For example, TNF-α may exert a more direct effect through binding to specific receptors expressed by DA neurons, such as the tumor necrosis factor receptor (TNFR). Upon TNF-α binding, the intracellular death domain of TNFR1 binds to the adaptor molecules TNFR1-associated protein with a death domain (TRADD) and FAS-associated protein with a death domain (FADD), then activate the apoptosis effector caspases [306]. In line with this possibility, the expression of TNF-α in the brain and CSF of PD patients are increased [307,308,309]. A polymorphism of the TNF gene has been reported in early onset PD patients [310]. In the SN of the PD patient, the TNFR1 level [311] and the activation of caspase-1, caspase-3, and caspase-8 [311,312,313] were all found significantly higher, while the percentage of FADD-immunoreactive DA neurons was decreased [306]. Mice deficient in both the TNFR1/2 (TNFR-DKO), but not the individual receptors, protect against MPTP-induced DA neurotoxicity [314,315,316]. Understood together, these evidence suggests that the TNF-α/TNFR pathway is very likely involved in the feedback of activated glia on DA neuron degeneration (Figure 3).

DA neurons are particularly vulnerable to oxidative stress. The reactive microglia could produce neurotoxic factors, especially the NADPH-oxidase-mediated release of ROS, and trigger DA neuron oxidative stress, which would then lead to α-syn nitration and cell death in PD models both in vitro and in vivo [180,191,238,239,317]. In the SN of human PD patients and MPTP-induced PD mice, NADPH-oxidase is upregulated and the NADPH-oxidase-defective mice showed less DA neuronal loss and protein oxidation [318,319]. Pro-inflammatory cytokines can also induce glial NO production [320], which may diffuse toward DA neurons and perform a deleterious role [321]. In support of this hypothesis, the level of nitrite was increased in the CSF of PD patients [322] and detectable nitrotyrosine was found in LBs [323]. Thus, the neuron-glia crosstalk forms a vicious cycle in PD, neuronal-released α-syn activates glia, which in turn leads to neurotoxic feedback on DA via producing pro-inflammatory factors and ROS [180] (Figure 3). Based on this, blocking glia activation might be neuroprotective. In fact, minocycline, an approved tetracycline derivative, mitigates MPTP- or mutant α-syn-induced DA neuronal loss, pathological α-syn accumulation, and motor dysfunction via inhibiting microglia activation and inflammation [324,325].

## 6. Ferroptosis in PD

### 6.1. Iron Deposition, α-Syn Aggregation, and Ferroptosis in PD

Iron homeostasis imbalance and accumulation in the SN are key clinical features of PD and ferroptosis [326]. Ferroptosis, initiated by abnormal iron metabolism and severe lipid peroxidation, leads to oxidative stress and cell death in an iron-dependent manner. It differs genetically, morphologically, and biochemically from other forms of programmed cell death such as apoptosis, necrosis, or autophagy. The cytological features of ferroptosis include cell volume shrinkage, smaller mitochondria size and cristae disappearance, and increased mitochondria membrane density and outer membrane rupture, while the nuclei maintain structural integrity. No plasma membrane rupture, cytoplasmic or organelle swelling, or apoptotic body formation was observed [327,328,329].

The PD patients postmortem study revealed the coexistence of iron with α-syn in midbrain LB [330]. Several other studies also show iron accumulates in the SN and colocalizes with α-syn, implying a link between iron deposition and α-syn aggregation [331]. α-syn is a metal-binding protein and iron binding enhances α-syn aggregation by generating conformational changes [332]. Furthermore, the 5′-UTR of α-syn mRNA has a unique RNA stem-loop with an iron responsive element (IRE) motif (CAGUGN) and iron regulatory protein (IRPs) can bind to the IRE motif of α-syn mRNA and block translation [333]. Iron, on the other hand, can reverse the translation inhibition of α-syn by IRP, hence iron accumulation can increase α-syn levels [334]. Recent research in human iPSC-derived neurons has shown that α-syn aggregation causes excessive calcium influx and ferroptosis by ROS production and oxidative damage [335]. Ferroptosis was also detected in other cellular and animal PD models, including MPP+-treated iPSC cells and MPTP-lesioned mice [336,337,338]. Furthermore, inhibiting ferroptosis with iron chelators or ferrostatin can ameliorate locomotor behavioral impairments and repair TH neuronal loss in MPTP-induced PD animal models [339]. Some clinical characteristics of PD are also known critical triggers of ferroptosis, including decreased glutathione (GSH) cystine/glutamate exchange transporter XcT [340,341], elevated lipid peroxidation products [342], *DJ-1* depletion, and so on [343].

### 6.2. Molecular Mechanism of DA Neuron Ferroptosis

**Figure 4:**
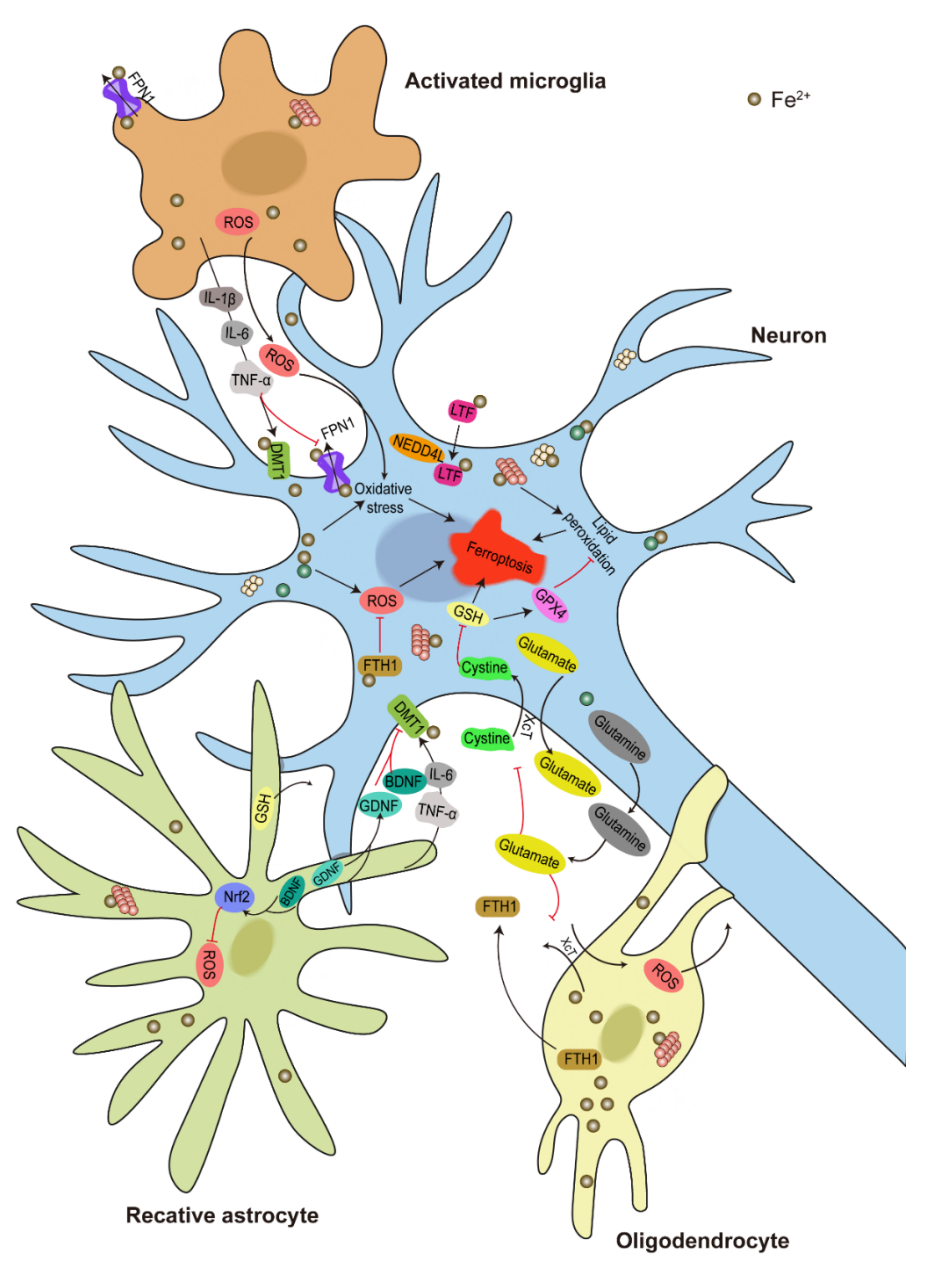
Glial activation in regulating DA neuron ferroptosis. Iron binding could enhance α-syn aggregation by generating conformational changes in neuron. Many genes have been involved in regulating ferroptosis. NEDD4L inhibits intracellular iron accumulation and ferroptosis through binding and degrades lactotransferrin (LTF). Glutathione peroxidase 4 (GPX4) suppresses lipid peroxidation and this requires GSH. The glutamine secreted from neurons can be converted into glutamate and increase extracellular glutamate content, which in turn inhibits XcT function and results in ferroptosis. All three types of glia could regulate DA neuron ferroptosis, in either promoting or inhibiting ways. Iron accumulation induces microglia activation, activated microglia promote pro-inflammatory factors release such as TNF-α, IL-6, IL-1β, and ROS production, all of which contributes to DA neuronal death. Inflammatory cytokines produced by active microglia and astrocytes could upregulate Divalent metal transporter 1 (DMT1) and downregulate ferroportin1 (FPN1), resulting in iron accumulation in neurons. Astrocytes can help to prevent iron overload in neurons, disruptions in astrocyte-neuron connections and insufficient Nrf2 activation may result in ferroptosis in neurons. Astrocytes transmit extra GSH to the neuronal region, replenishing the antioxidant dysfunction in neurons during ferroptosis. Active astrocytes also release BDNF and GDNF to inhibited iron uptake by reducing DMT1 expression. Astrocytes and oligodendrocytes regulate glutamate levels in the synaptic cleft by regulating GSH production and suppressing neuronal XcT. Oligodendrocytes secrete FTH1 into the extracellular space to protect neurons from oxidative injury.

A study in PD patients found that iron levels in a single SN DA neuron can rise by almost two times and that this may be related to the accelerated deleterious formation of α-syn aggregates [344]. In patients’ small-molecule-derived neuronal precursor cells with *SNCA* triplication, elevated α-syn expression increases lipid peroxidation and ferroptosis. Reducing α-syn expression in DA neurons leads to ferroptosis resistance due to a reduction in ether-linked phospholipids, which is required for ferroptosis [345]. Many genes have been involved in regulating ferroptosis (Figure 4). For example, a study in human pancreatic cancer cells (PANC1) have screened 571 UPS-related genes and identified NEDD4-like E3 ubiquitin protein ligase (NEDD4L) as a novel suppressor of ferroptosis. NEDD4L binds to and degrades lactotransferrin (LTF), an iron-binding transport protein, thus inhibits intracellular iron accumulation and ferroptosis [346]. Studies in a 6-OHDA-induced rat PD model and 6-OHDA-treated PC-12 cells found ferritin heavy chain 1 (FTH1) could inhibit ferroptosis through ferritinophagy, a selective form of autophagy [347]. Divalent metal transporter 1 (DMT1) overexpression has been linked to intracellular iron accumulation and neurotoxicity in human A53T α-syn transgenic PD mice [348]. Glutathione peroxidase 4 (GPX4) is a crucial enzyme engaged in lipid peroxidation, it can degrade small-molecule peroxides and some lipid peroxides, thus suppressing lipid peroxidation [349]. GSH is required for the GPX4 lipid repair function, when GSH is exhausted, GPX4 is inactivated, resulting in membrane lipid ROS buildup and ferroptosis [350]. The glutamine secreted from neurons can be converted into glutamate and increase extracellular glutamate content, which in turn inhibits XcT function and results in ferroptosis [351]. Numerous studies have concentrated on figuring out how Nrf2 affect ferroptosis, because Nrf2 play a major role in regulating the metabolism of iron, lipids, and GSH [352]. Additionally, Nrf2 appears to be mostly localized to nuclei in the SN of PD patients [353]. Thus, it might be another molecule that connect PD and ferroptosis.

### 6.3. Glial Activation in Regulating DA Neuron Ferroptosis

Glia can execute ferroptosis inhibiting and neuroprotective actions. Astrocytes have a high capacity for iron storage and are specialized for iron export, this can help to prevent iron overload in neurons [354]. As a result, disruptions in astrocyte-neuron connections and insufficient Nrf2 activation in astrocytes may result in ferroptosis in neurons, particularly DA neurons [355]. Astrocytes have the potential to transmit extra GSH to the neuronal region, replenishing the antioxidant dysfunction in neurons during ferroptosis [356]. Active astrocytes also release BDNF and GDNF to inhibit iron uptake by reducing DMT1 expression and decrease iron accumulation in neurons [357]. Furthermore, astrocytes and oligodendrocytes effectively regulate glutamate levels in the synaptic cleft by regulating GSH production and suppressing neuronal XcT [358]. Oligodendrocytes, the most iron-rich cells in the brain, require adequate iron to create myelin sheaths [359]. Studies have revealed that oligodendrocytes are particularly vulnerable to oxidative stress, iron load increases the risk of oligodendrocyte destruction, and PD patients experience demyelinating alterations in the brain [360]. Oligodendrocytes could secrete FTH1 into the extracellular space to protect neurons from oxidative injury [361]. Understood together, all three types of glia could regulate neuron ferroptosis, in either promoting or inhibiting ways (Figure 4).

On the other hand, both iron accumulation and glial activation are important features of PD pathology, they can accelerate DA neuron degeneration by mutually influencing one another and act as “partners in crime” [362]. Glial activation results in iron imbalance, which in turn exacerbates microglial activation, then causes more serious pathological symptoms [363]. Research in cultured mouse microglia showed that iron accumulation could cause microglia to polarize to the M1 phenotype, induce microglial dysfunction and reduce phagocytosis [364]. Additionally, iron accumulation-induced microglia activation could elevate ferritin expression, promote pro-inflammatory factors release such as TNF-α, IL-6, and IL-1β, and induce ROS production, all of which contributes to DA neuronal death [365,366]. Inflammatory cytokines produced by active microglia and astrocytes also could upregulate DMT1 and downregulate ferroportin1 (FPN1), resulting in iron accumulation in neurons [367] (Figure 4). Interestingly, many studies have demonstrated that microglia are resistant to ferroptosis. M1 microglia are more resistant to ferroptosis than M2 microglia, most likely due to their extensive expression of Nrf2 and enrichment of iNOS/NO [368].

### 6.4. Iron Transport between Neurons and Glia

Since iron deposition are found in both glia and neurons, an obvious question is raised. Can iron transfer between neurons and glia similar to α-syn? Iron transmission between cells through extracellular vesicles have been well studied, but direct transfer between neurons and glia have not been detected yet. Iron metabolism-regulating proteins such as iron-rich ferritin can transmit between cells via the secreted vesicles, such as exosomes, and this iron export promotes ferroptosis resistance in mammary epithelial and breast carcinoma cells [369,370]. Furthermore, transferrin (Tf) and transferrin receptor (TfR) were shown to be transferred between cells through TNTs [371]. As mentioned in the preceding text, iron can bind to α-syn [332], meaning it is possible that iron can transfer between neurons and glia via binding to α-syn, another potential route of glia in regulating neurodegeneration, and the further mechanism warrants investigation.

## 7. Prospective of PD Therapy

### 7.1. Classical Treatments of PD

To date, there is no cure for PD. Considering the crucial role of α-syn in PD pathogenesis, multiple therapeutic strategies are developed to target α-syn, including decrease gene expression, stabilize physiological conformation, inhibite aggregation, increase clearance, and so on (Table 2) [372]. Clenbuterol and salbutamol, two β2-adrenoreceptor (β2AR) agonists, have been shown to reduce α-syn expression by modulating the activity of histone deacetylase (HDAC) at gene promoter and enhancer regions. These modifications were found to be neuroprotective in PD patient-derived cell lines and an MPTP-induced mouse model [373]. Yet, these β2AR agonists currently are not approved by the FDA and could aggravate cardiovascular disease [374]. The monoclonal antibody ABBV-0805 (mAb47) has a relatively low affinity for α-syn monomers and a strong selectivity for α-syn aggregates [375]. An in vivo study demonstrated that mAb47 treatment could reduce α-syn aggregates in a dose-dependent manner and extend the lifetime of α-syn transgenic mice [376]. However, the clinical significance of mAb47 is still unknown [375]. Understood together, additional populations and clinical trials are still needed to determine the effects of drugs targeting α-syn.

As aforementioned, medical therapy frequently results in side effects such as depression [377]. Additional therapies are developed to relieve symptoms and maintain the qualities of patients’ lives. Surgical intervention such as deep brain stimulation (DBS) in particular is a well-established nondestructive treatment [378], but it entails surgical risk and is just palliative [379]. Gene therapy is another promising non-ablative treatment that involves stereotactically delivering therapeutic genes via viral vectors into the precise places in patients’ brains, so that they can enter cells and produce the appropriate gene products over time and locally [380]. However, none of these tried-and-true medicinal or surgical procedures can stop or reverse DA neuron loss over time. One effective way for DA neuron rescue is cell therapy, numerous different cell types have been employed for cell transplantation in PD patients over the past 40 years [381,382,383], but the host immune response to the transplanted tissue is one of the most serious problems. Endogenous neurogenesis in the adult brain can be thought as potential neuronal repair treatment. Neural stem/progenitor cells (NPCs), which are self-renewing multipotent cells that give rise to neurons and glia in nervous systems, are now being elucidated by several research groups for their important role in PD [384]; as a result, they may have the capacity to replace lost DA neurons, but this is still preliminary. Thus, methods for producing immune-tolerable, genetically stable cells at a sustained clinical-grade level are in urgent need [385].

### 7.2. Glia as Potential Target for Early PD Treatment

Current PD treatments did not slow down the disease progression, while motor symptoms usually occur at the late stage of PD, after 50% of the DA neurons have been lost [386], so it is critical to find early-stage biomarkers. As previously mentioned, glial cells are important participants in early onset PD and its progression [387]. Glia-released inflammation, cytokines, and chemokines serve as potential early-stage biomarkers [388,389]. Particularly, TNF-α, Il-β, and IL-6 were reported as possible PD biomarkers and their serum concentrations correlate with PD severity [390,391,392]. Higher levels of serum TNF-α was a characteristic of PD patients with symptoms such as cognition, depression, and disability [393,394]. As a result, targeting glial cells may provide a future avenue for developing new treatments during the prodromal phase of PD (Table 2). As previously mentioned, minocycline plays a wide range of neuroprotective roles in PD animal models by inhibiting microglia-related inflammation [324,325,395,396,397] and it serves as a potential therapeutic agent for PD treatment. Nevertheless, minocycline is reported to play non-beneficial or even deleterious effects. Minocycline could attenuate striatal tyrosine hydroxylase in GDNF+/− mice, but not methamphetamine-induced tyrosine hydroxylase reduction [398]. Clinical trials in early PD patients showed minocycline are futile in slowing down PD progression [399,400]. Studies also found minocycline could exacerbate the toxicity of MPTP to DA neurons [401,402]. Low and middle concentrations of minocycline protected knockdown transgenic parkin *Drosophila* against paraquat toxicity, but high concentrations aggravated the toxic effect [396]. Thus, the effect of minocycline may vary depending on the disease model or administration dose. In addition, it has been demonstrated that the drug Glatiramer acetate reduces microglial activation by promoting M2 transition. Glatiramer acetate-treated MPTP mice effectively reversed clinical motor dysfunction, as well as the pathology features including TH, IBA1, and BDNF expression [403].

Recently, reprogramming glial cells into DA neurons has provided a new path for PD treatment [404]. The effective reprogramming of midbrain astrocytes into functional DA neurons in a mouse PD model marks an important breakthrough, because such a method can replace a major portion of the lost DA neurons and greatly avoid the host immune response. Yet, it is still short of a developed method and whether reprogramming works in middle-aged and elderly animal midbrains is still unknown. Various therapies have been developed based on astrocyte transplantation and pharmacological targeting astrocytes (Table 2). Human astrocytes can be obtained from primary sources or by differentiating NPCs or iPSCs [405]; once the stem cells differentiate into astrocytes, they can be transplanted to replace defective cells or to help the existing neuronal cells survive [406]. Even though the research on glia treatment in PD is incomparable to neurons, it is currently drawing more and more attention.

### 7.3. Ferroptosis Inhibitors in PD Treatment

The critical role of ferroptosis in PD suggests that ferroptosis modulators could also be used as possible therapies (Table 2). The treatment of ferroptosis inhibitors FTH1 in 6-OHDA-induced PD rats were reported to relieve motor symptoms [347]. The research on animals has demonstrated that iron chelators that can cross the BBB have distinct protective effects on PD mice [407]. Importantly, a clinical trial of the iron chelating drug deferiprone (DFP) demonstrated that iron repellent treatment could reduce iron levels and alleviate motor symptoms in patients with early PD [407]. While more PD therapies targeting glia and ferroptosis are blooming, further mechanism investigations are still needed.

**Table 2:** Representative approaches for PD treatment.

Therapy	Mechanism	Drugs or Methods	Weakness or Note
Targeting α-syn	Decrease α-syn expression	Clenbuterol, Salbutamol	A potential to aggravate cardiovascular disease [374].
	Inhibit α-syn aggregates	NPT200-11	Cannot pass through the BBB or might cause even serious poisoning [408].
	Reduce α-syn aggregates	ABBV-0805	Additional populations and clinical trials are still needed to determine the effects [375].
	Increase α-syn degradation	Nilotinib	The lack of efficacy on motor outcomes indicated that nilotinib had no clinical benefits [409].
	Reduce α-syn spreading	BIIB054 (cinpanemab)	Lack of efficacy data and PK data in participants [410].
Targeting glia	Inhibit microglia activation	Minocycline Amantadine	Lack of mechanical understanding on microglial activation [411].
	Promote anti-inflammation factors in microglia	Cyclin-AMP PPAR-γ agonists	
	Deliver chemicals specifically to astrocytes	Peptide-Based Delivery/Viral Delivery	Still unable to specifically deliver drugs to distinct astrocyte subpopulations [412].
	Deliver protect genes such as *GDNF* to CNS	AAV delivery	Recent preclinical and clinical findings point to significant immune reactions and neuroinflammation associated with vectors [413].
	Astrocyte reprogramming	Astrocyte	Teratoma formation and immune reaction [414].
Targeting ferroptosis	A medication that chelates iron and is used to treat iron overload	Deferiprone	Waiting for clinical trial in PD patients [415].
	Reduce ALOX12 levels and reverses paraquat-induced ferroptosis	Fer-1	Only show effect in a small part of neurons [338].

## 8. Conclusions

In the past decades, extensive neuron-glia crosstalk was revealed in regulating the pathogenesis of α-syn in PD onset and progression, proving the critic roles of glial cells than we previously realized; as a result, glial activation and inflammation are emerging as new features of PD pathology. However, many intriguing questions still need to be investigated, such as how the pro-inflammatory cytokines and neurons/glia recognize and regulate each other? How neuroprotective glia transit to neurotoxic glia? New approaches such as single-cell transcriptomics, single-cell proteomics, and proteomics-based functional studies could be applied to tailor stage-specific neuron/glia signatures in PD; this will not only help us to understand the etiology of PD but also improve the therapeutic efficiency in targeting glia.

## Data Availability

Not applicable.
